# Safety and Tolerance of *Bifidobacterium longum subsp. Infantis* YLGB-1496 in Toddlers with Respiratory Symptoms

**DOI:** 10.3390/nu17132127

**Published:** 2025-06-26

**Authors:** Pin Li, Mageswaran Uma Mageswary, Fahisham Taib, Thai Hau Koo, Azianey Yusof, Intan Juliana Abd Hamid, Hua Jiang, Min-Tze Liong, Adli Ali, Yumei Zhang

**Affiliations:** 1 Department of Nutrition and Food Hygiene, School of Public Health, Peking University Health Science Center, Beijing 100191, China; 2 School of Industrial Technology, Universiti Sains Malaysia, Gelugor 11800, Malaysia; 3 Pediatric & Palliative Care, Hospital Pakar Universiti Sains Malaysia, Kota Bharu 16150, Malaysia; 4 Kepala Batas Health Clinic, Kepala Batas 13200, Malaysia; 5 Cluster of Regenerative Medicine, Advanced Medical & Dental Institute, Universiti Sains Malaysia, Bertam 13200, Malaysia; 6 School of Nursing, Peking University, Beijing 100191, China; 7 Department of Pediatrics, UKM Medical Centre, Faculty of Medicine, Universiti Kebangsaan Malaysia, Kuala Lumpur 56000, Malaysia

**Keywords:** *B. infantis*, probiotics, toddlers, gastrointestinal symptoms

## Abstract

**Objective:** The aim of this study was to examine the safety and tolerance of *Bifidobacterium longum subsp. infantis* YLGB-1496 (*B. infantis* YLGB-1496) in toddlers with respiratory illness. **Methods**: In this randomized controlled trial, 120 toddlers with respiratory illness were randomly assigned to the probiotic (YLGB-1496) or control group for a 12-week intervention. Follow-up examinations were conducted at baseline (week 0) and at weeks 6 and 12 of the intervention. Toddlers’ height and weight were measured by trained personnel, and defecation characteristics and gastrointestinal symptoms were recorded by parents or guardians. Stool samples were collected to determine the fecal pH, fecal calprotectin (FC) concentration, and fecal α1-antitrypsin (AAT) concentration. **Results**: A total of 115 toddlers completed the 12-week intervention (58 in the YLGB-1496 group and 57 in the control group). The height-for-age Z score (HAZ) in the YLGB-1496 group was significantly greater than that in the control group (*p* = 0.006). The weight-for-age Z score (WAZ) in the YLGB-1496 group increased between weeks 6 and 12, whereas the WAZ in the control group continuously decreased during the intervention. No differences in the frequency or consistency of defecation between the groups were observed. Toddlers in the YLGB-1496 group had lower incidences of poor appetite, nausea, vomiting, stomachache, lower abdominal pain, diarrhea, and dehydration (*p* < 0.05) but higher fecal AAT concentrations (*p* = 0.008) than did those in the control group. No differences in the fecal pH or FC concentration were observed between the groups. **Conclusions**: *B. infantis* YLGB-1496 demonstrated excellent safety and tolerability in toddlers and effectively reduced the gastrointestinal discomfort associated with respiratory illnesses.

## 1. Introduction

*Bifidobacterium* was first isolated from the feces of breastfed infants by Tissier [1]. Substantial clinical evidence supports the use of *Bifidobacterium* as a therapeutic option for both preterm and healthy full-term infants [2]. These bacteria are inherently vital components of a healthy infant gut microbiome, thus indicating significant potential for clinical application. While the majority of *Lactobacillus* and *Bifidobacterium* strains are recognized as probiotics, *Bifidobacterium longum subsp. Infantis* (*B. infantis*) may be particularly important during early life. Previous studies have demonstrated that *B. infantis* supplementation promotes the development of the immune system, intestinal maturation, gut microbiome balance, and digestive system absorption in infants and young children [3,4,5,6].

Research indicates that *B. infantis* increases the proliferation and differentiation of intestinal epithelial cells and stem cells. By modulating the expression of transmembrane proteins [7] and strengthening tight junctions between epithelial cells [8], *B. infantis* improves the function of the intestinal mucosal barrier [9]. In the assessment of intestinal maturity in infants and young children, fecal calprotectin (FC) and α1-antitrypsin (AAT) are commonly used biomarkers. Since AAT is neither degraded by digestive enzymes nor reabsorbed by the intestines, its levels correlate positively with the extent of serum protein loss in the gut, making it a useful indicator for evaluating gastrointestinal development and inflammatory status [10]. Studies have confirmed that *B. infantis* reduces FC concentrations in infants [11], suggesting that probiotics may improve intestinal barrier function by modulating gut permeability.

Several intervention trials have demonstrated that specific strains of *B. infantis* can modulate stool consistency, reducing the incidence of both constipation and diarrhea in infants and toddlers [12,13].

This manuscript presents a secondary analysis of data derived from our previously published randomized controlled trial (NCT05794815) [14], which established the efficacy of *Bifidobacterium longum subsp. infantis* YLGB-1496 (*B. infantis* YLGB-1496) in reducing respiratory symptoms in toddlers. However, the safety profile, tolerability, and specific impacts of this probiotic intervention on growth metrics and gastrointestinal (GI) health in toddlers with respiratory illnesses remained unexplored in that primary report. Therefore, the specific aims of this secondary analysis are: (a) to rigorously evaluate the safety and tolerability of *B. infantis* YLGB-1496 in toddlers (aged 1–3 years) experiencing respiratory illnesses and (b) to explore its effects on the intestinal health of this specific pediatric population. This work thus addresses critical gaps by focusing on these previously unreported variables from the original RCT dataset.

## 2. Materials and Methods

### 2.1. Study Design

**Figure 1:**
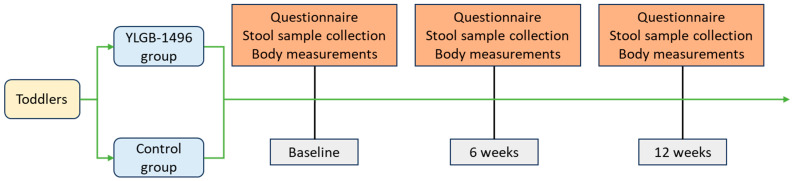
Experimental Procedure.

Briefly, 120 toddlers with at least 2 kinds of respiratory symptoms were randomized 1:1 to receive *B. infantis* YLGB-1496 1 × 10^10^ colony-forming units (CFUs)/day or placebo (maltodextrin) in a 12-week intervention (Figure 1). According to previous studies, a high dose of *B. infantis* (1.8–2.8 × 10^10^ CFUs/day) is safe and well-tolerated in healthy infants [13,15].

### 2.2. Outcomes and Measurements

The outcomes of this analysis were physical development, gastrointestinal symptoms, defecation characteristics (stool hardness and defecation frequency), fecal pH, and FC and AAT concentrations.

#### 2.2.1. Questionnaires

All questionnaires used in the study were validated for reliability and validity and translated into Malay [16]. Questionnaires primarily consisted of the following sections:

(a) Sociodemographic questionnaire.
(b) Stool characteristics: Stool consistency (evaluated using the Bristol Stool Scale (BSS)) and frequency.
(c) Gastrointestinal health: Frequency of adverse gastrointestinal symptoms (poor appetite, nausea, vomiting, stomachache, anal discomfort, diarrhea, and dehydration).
(d) Dietary intake: Consumption rates of breast milk, formula, yogurt, other dairy products, grains and tubers, vegetables, fruits, protein sources (meat, eggs, and seafood), beverages, snacks, and various nutritional supplements.
(e) Adverse events (AEs) and serious adverse events (SAEs).

#### 2.2.2. Body Measurements

At weeks 0, 6, and 12 of the intervention, professionally trained nurses, who had undergone uniform training, measured the height and weight of the infants using standardized measuring beds and scales. World Health Organization (WHO) Anthro software (Version 3.2) was used to calculate the growth and development Z scores of the infants on the basis of the onsite measurements of their height/length and weight data. These Z scores included the height-for-age Z score (HAZ), weight-for-age Z score (WAZ), weight-for-height Z score (WHZ), and body-mass-index-for-age Z score (BAZ).

#### 2.2.3. Sample Collection and Analysis

Stool samples were collected at weeks 0, 6, and 12 of the intervention. The pH of the stool samples was measured using a solid and semisolid pH meter. The concentrations of AAT and FC in the stool were determined using enzyme-linked immunosorbent assay (ELISA) kits (Sunlong Biotech, Hangzhou, China).

### 2.3. Statistical Analysis

All analyses followed the intention-to-treat principle, consistent with the primary trial [14]. Continuous variables are presented as the mean value ± the standard deviation (mean ± SD) unless otherwise stated. Categorical variables are presented as frequencies and percentages (n, %). Differences in continuous variables between groups or across different time points were compared using Student’s *t*-test. Differences in count variables were analyzed using Poisson regression.

A mixed-effects model was employed to analyze the effects of intervention methods and intervention duration on various outcome measures. Baseline values, group, intervention time, and the interaction term between group and time were included as fixed effects, whereas individuals were treated as random effects. Confounding factors that differed between the two groups were considered in different models, and linear mixed-effects models were constructed. If the interaction term was significant, post hoc tests were conducted; if the interaction term was not significant, it was removed from the model for further analysis. For count variables as outcome measures, a generalized mixed-effects model with family = Poisson was used for analysis. All tests were two-sided, with *p* < 0.05 considered to indicate statistical significance, and were performed via R software version 4.2.3.

## 3. Results

### 3.1. Baseline Characteristics

Among the 120 screened participants, 63 were randomized to the YLGB-1496 group and 57 were randomized to the control group. Five participants in the YLGB-1496 group withdrew prior to intervention initiation. Consequently, 115 participants were ultimately included in the analysis (58 in the YLGB-1496 group and 57 in the control group); see the CONSORT flow diagram in the published paper [14]. The cesarean section rate was marginally higher in the YLGB-1496 group than in the control group (*p* = 0.049). Statistically significant between-group differences were observed in family caregiving methods (*p* = 0.003). Significant intergroup differences were not observed for any other outcomes.

At baseline, the breastfeeding rate was significantly higher in the YLGB-1496 group than the placebo group (*p* = 0.001). Additionally, vegetable consumption rates in the YLGB-1496 group remained lower than those in the control group at both baseline and week 12 (*p* < 0.05). No statistically significant differences in the consumption rates of other food categories or nutritional supplements were observed between the groups (complete data are available in the published paper [14]).

### 3.2. Growth and Development

**Figure 2:**
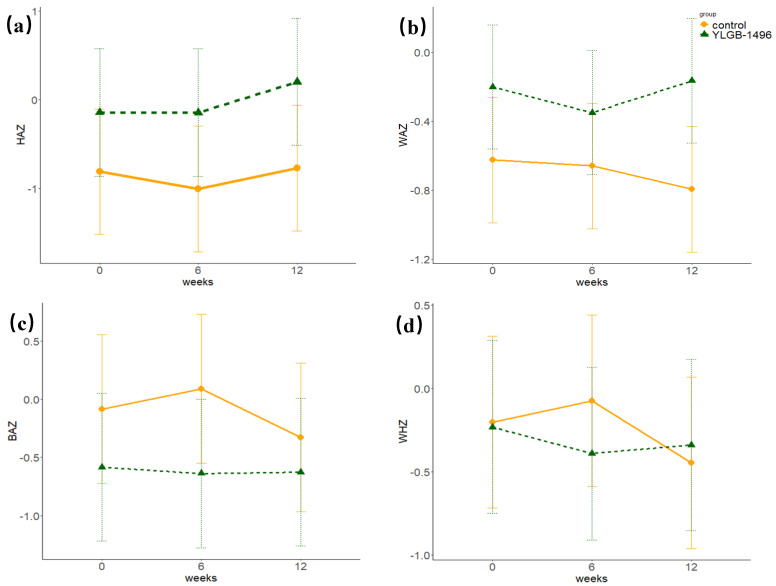
Growth and Development Z Scores in Toddlers (estimated marginal means and contrasts): (**a**) The height-for-age Z score (HAZ), (**b**) The weight-for-age Z score (WAZ), (**c**) The body-mass-index-for-age Z score (BAZ), and (**d**) The weight-for-height Z score (WHZ).

The growth and development Z scores for the children in both groups during the intervention period are presented in Table 1. The emmeans package was employed to obtain estimated marginal means and contrasts of Z score changes in growth parameters (Figure 2). After adjusting for confounders, including dietary factors, through linear mixed-effects models, children in the YLGB-1496 group demonstrated significantly greater HAZ than did those in the control group. Additionally, WAZ in the YLGB-1496 group moderately increased from 6–12 weeks, whereas the WAZ in the control group continuously decreased throughout the intervention period, indicating divergent temporal trends between the groups (Figure 2b). No significant differences in the WHZ or BAZ were observed between the groups.

### 3.3. Defecation Characteristics

**Figure 3:**
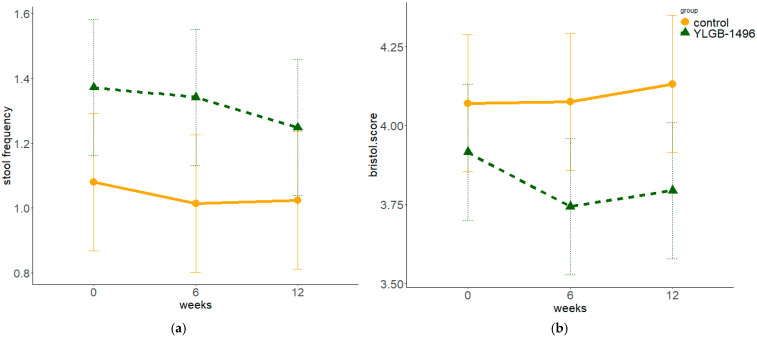
Stool Frequency and Consistency in Toddlers (estimated marginal means and contrasts): (**a**) Stool frequency per day, (**b**) Bristol Stool Scale scores.

A summary of the changes in stool consistency and daily defecation frequency during the intervention is presented in Table 2 and Figure 3. Both groups maintained a daily defecation frequency of 1–2 times, with BSS scores of approximately 4 points. Although the YLGB-1496 group had a marginally greater defecation frequency and slightly lower BSS scores than the control group did, these differences were not significant in either the intergroup comparisons or the temporal analyses after adjusting for baseline values and dietary intake.

### 3.4. Gastrointestinal Symptoms

At baseline, some children had gastrointestinal symptoms, including poor appetite and diarrhea alongside respiratory discomfort. A summary of the incidence and occurrence rates of various gastrointestinal adverse symptoms during the intervention is presented in Table 3. In the YLGB-1496 group, the incidence rates of most gastrointestinal symptoms progressively decreased over time, whereas in the control group, incidence rates remained relatively stable. Generalized mixed-effects model analysis (Table 4) revealed significantly lower incidence rates of poor appetite, nausea, vomiting, abdominal pain, intestinal colic, diarrhea, and dehydration symptoms in the YLGB-1496 group than in the control group.

### 3.5. Biochemical Indicators

After adjusting for potential confounders, linear mixed-effects models revealed significantly higher AAT concentrations in the YLGB-1496 group. No significant intergroup differences were detected in stool pH or FC concentrations throughout the intervention period (Table 5).

## 4. Discussion

In our previous study, the effects of *B. infantis* YLGB-1496 intervention on respiratory symptoms in young children were elucidated [14]. Building on these findings, in this secondary analysis, we focused on evaluating the safety and tolerability of the intervention, particularly its impact on growth, development, and intestinal health.

In this study, we found that the HAZ in the YLGB-1496 group was significantly greater than that in the control group. Additionally, the WAZ in the YLGB-1496 group moderately increased during the later stages of the intervention, whereas that in the control group continuously decreased. Given the strong metabolic capacity of *B. infantis* for human milk oligosaccharides (HMOs), it is hypothesized that this strain promotes the production of short-chain fatty acids in the infant gut, thereby improving digestion and absorption and potentially regulating physical growth [17,18]. Animal studies have demonstrated that *B. infantis* CCFM1269 promotes osteoblast differentiation and the expression of bone-formation-related genes by modulating the gut microbiota and metabolites, significantly promoting bone formation and increasing the length of the femur and tibia [19]. Population trials have also revealed faster HAZ growth in infants receiving *B. infantis* supplements [20]. However, a meta-analysis on the impact of *B. infantis* on infant growth revealed that, while *B. infantis* supplementation does not negatively affect growth, it also does not significantly promote growth [21]. Furthermore, infants receiving probiotic supplementation alone showed greater increases in length and weight than those receiving synbiotics [22].

Stool frequency and consistency were assessed among the participants. Although the YLGB-1496 group presented slightly greater stool frequency and lower BSS scores than the control group, these differences were not statistically significant after adjustments were made for potential confounders. Previous studies on other *B. infantis* strains have revealed that *B. infantis* supplementation reduces stool softness in infants and that higher doses may increase the frequency of pasty or watery stools, whereas lower doses tend to reduce the number of formed stools and increase the number of soft, unformed stools [15,23]. A randomized controlled trial in healthy full-term infants revealed that *B. infantis* supplementation significantly reduced the incidence of both diarrhea and constipation [12]. These findings suggest that *B. infantis* may have a bidirectional regulatory effect on stool characteristics, adjusting stool frequency and consistency toward an optimal range. In this study, the mean BSS score of toddlers was approximately 4, indicating soft, formed stools, which may explain the lack of significant differences observed.

The intervention significantly reduced gastrointestinal discomfort, including poor appetite, nausea, vomiting, stomachache, anal discomfort, and diarrhea. These results are consistent with findings from trials involving other *B. infantis* strains [24]. Compared with lower doses, higher doses of probiotics are associated with faster symptom relief [15]. Similar conclusions were drawn from a study evaluating infant formula supplemented with *B. infantis* [25]. Meta-analyses have indicated that probiotics positively impact irritable bowel syndrome, abdominal pain, and bloating in children [26] and provide moderate protection against antibiotic-associated diarrhea, reducing its duration [27]. However, some studies have revealed no significant differences in gastrointestinal symptom outcomes between different probiotic doses [28].

The gastrointestinal tract of infants and young children is still developing, with immature tight junctions between intestinal epithelial cells and relatively high intestinal permeability. In this study, the concentration of AAT was significantly greater in the YLGB-1496 group than in the control group. No significant effects of *B. infantis* YLGB-1496 on the FC concentration or fecal pH were observed. The impact of probiotics on fecal pH appears to depend on the intervention dose and population, with higher doses and exclusively breastfed infants often exhibiting more pronounced and sustained reductions in fecal pH [15,17].

Biomarkers for assessing gastrointestinal maturity in infants and young children are limited, and previous findings have been inconsistent. Animal studies have shown that *B. infantis* FJSYZ1M3 improves the integrity of intestinal tight junctions [29]. Some animal studies have revealed that neither probiotics nor prebiotics alone affect FC concentrations [30], although *B. longum* has been shown to reduce intestinal damage in rats with inflammatory bowel disease [31]. A randomized controlled trial in adults with gastrointestinal symptoms using *B. longum* CECT7347 revealed no significant differences in FC concentrations between groups [32]. Previous studies have shown that mixed probiotics can reduce intestinal AAT concentrations [10,33]. However, few studies have demonstrated that *B. infantis* supplementation lowers AAT concentrations in infants and young children. Conversely, a study in which high and low doses of *B. infantis* were administered to newborns revealed that both doses increased AAT concentrations, with more pronounced effects in exclusively breastfed infants [15]. Data indicate that breastfed infants have higher AAT concentrations than formula-fed infants do [34], which may partially explain these findings. The unexpected elevation of AAT in the YLGB-1496 group warrants critical interpretation, as it may indicate mucosal stimulation rather than inflammatory pathology. Meanwhile, recently, AAT has been shown to promote tissue remodeling and inflammatory resolution [35,36]. Given the consistently low baseline AAT concentrations observed in children with respiratory illnesses throughout this cohort, these elevated values do not support the conclusion that *B. infantis* YLGB-1496 supplementation increased intestinal permeability.

The main strength of this study is that it is the first in which the safety and tolerance of *B. infantis* YLGB-1496 application in toddlers were evaluated. We measured changes in FC and AAT concentrations while also considering the influence of dietary factors during data analysis. However, this study has notable limitations. The incidence of gastrointestinal symptoms was primarily self-reported by parents, which may have introduced reporting or recall bias into the statistical results. In addition, group differences were observed in baseline characteristics such as delivery mode and primary caregiving methods. While these factors were included as covariates in our multivariate regression models to mitigate confounding, they may still exert some influence on the observed intervention effects. Ethical approval mandated exclusive use of non-invasive fecal sampling. However, fecal biomarker levels are substantially influenced by environmental factors, limiting their representativeness and potentially obscuring intervention effects. To determine the effect and value of *B. infantis* YLGB-1496 in clinical practice, more studies with serum biomarkers, larger sample sizes, different probiotic doses, and longer interventions are still needed.

## 5. Conclusions

*B. infantis* YLGB-1496 demonstrated excellent safety and tolerability in toddlers. *B. infantis* YLGB-1496 effectively reduced gastrointestinal discomfort associated with respiratory illnesses and promoted intestinal health.

## Figures and Tables

**Table 1:** Effect of YLGB-1496 on Growth and Development Z Scores in Toddlers (Mean ± SD).

Variables	Control	YLGB-1496	*p * ^a^	Model ^b^		*p * ^c^	
					Week	Group	Week × Group
HAZ							
Baseline	−0.81 ± 2.78	−0.14 ± 2.49	0.184	1	0.007	0.165	0.820
6 weeks	−1.00 ± 2.91	−0.14 ± 2.48	0.096	2	0.007	0.075	
12 weeks	−0.77 ± 3.01	0.20 ± 2.58	0.069	3	0.007	0.006	
WAZ							
Baseline	−0.63 ± 1.54	−0.20 ± 1.48	0.135	1	0.194	0.162	0.029
6 weeks	−0.66 ± 1.37	−0.35 ± 1.38	0.230	2	0.194	0.243	
12 weeks	−0.80 ± 1.35	−0.17 ± 1.24	0.010	3	0.194	0.329	
BAZ							
Baseline	−0.08 ± 2.12	−0.58 ± 2.66	0.267	1	0.382	0.514	0.068
6 weeks	0.09 ± 2.36	−0.64 ± 2.52	0.116	2	0.379	0.414	
12 weeks	−0.33 ± 2.55	−0.63 ± 2.46	0.524	3	0.380	0.194	
WHZ							
Baseline	−0.20 ± 1.89	−0.23 ± 1.95	0.937	1	0.126	0.620	0.097
6 weeks	−0.07 ± 2.00	−0.39 ± 1.92	0.397	2	0.126	0.530	
12 weeks	−0.44 ± 2.14	−0.34 ± 1.84	0.781	3	0.126	0.293	

SD: standard deviation; HAZ: height-for-age Z score; WAZ: weight-for-age Z score; WHZ: weight-for-height Z score; and BAZ: body-mass-index-for-age Z score. ^a^ *p* value obtained via Student’s *t*-test. ^b^ Model 1: adjusted for baseline values, time, group, and interaction terms comprising time and group; Model 2: additionally adjusted for delivery and primary caregiver to correct for differences between groups; Model 3: additionally adjusted for breastmilk and vegetable intake during the intervention. ^c^ *p* values for the intervention were obtained from the linear mixed model.

**Table 2:** Effect of YLGB-1496 on Defecation Frequency and Stool Consistency in Study Participants (Mean ± SD).

Variables	Control	YLGB-1496	*p * ^a^	Model ^b^	*p * ^c^		
					Week	Group	Week × Group
Defecation frequency (times/day)							
Baseline	1.08 ± 0.82	1.37 ± 0.96	0.082	1	0.194	0.253	0.110
6 weeks	1.01 ± 0.72	1.34 ± 0.83	0.026	2	0.194	0.448	
12 weeks	1.02 ± 0.71	1.25 ± 0.79	0.115	3	0.194	0.211	
Bristol Stool Scale scores							
Baseline	4.07 ± 1.02	3.92 ± 0.85	0.377	1	0.214	0.018	0.942
6 weeks	4.07 ± 0.94	3.74 ± 0.51	0.021	2	0.214	0.073	
12 weeks	4.13 ± 1.02	3.79 ± 0.47	0.024	3	0.214	0.232	

SD: standard deviation. ^a^ *p* value obtained via Student’s *t*-test. ^b^ Model 1: adjusted for baseline values, time, group, and interaction terms comprising time and group; Model 2: additionally adjusted for delivery and primary caregiver to correct for differences between groups; Model 3: additionally adjusted for breastmilk and vegetable intake during the intervention. ^c^ *p* values for the intervention were obtained from the linear mixed model.

**Table 3:** Incidence of Gastrointestinal Symptoms during the Intervention.

Variables	Weeks	Control		YLGB-1496		IRR (95% CI)	*p * ^a^
		N	IR (SE)	N	IR (SE)		
Poor appetite	0	43	0.754 (0.165)	30	0.517 (0.128)	0.686 (0.426, 1.088)	0.113
	6	43	0.754 (0.165)	6	0.103 (0.053)	0.137 (0.052, 0.298)	<0.001
	12	42	0.737 (0.165)	0	0 (0)	——	——
Nausea	0	15	0.263 (0.073)	13	0.224 (0.065)	0.852 (0.399, 1.793)	0.672
	6	14	0.246 (0.058)	4	0.069 (0.042)	0.281 (0.080, 0.783)	0.025
	12	13	0.228 (0.056)	0	0 (0)	——	——
Vomiting	0	18	0.316 (0.076)	19	0.328 (0.131)	1.037 (0.542, 1.991)	0.911
	6	16	0.281 (0.060)	6	0.103 (0.047)	0.369 (0.132, 0.896)	0.037
	12	15	0.263 (0.059)	2	0.034 (0.024)	0.131 (0.021, 0.464)	0.007
Stomachache	0	23	0.404 (0.082)	24	0.414 (0.085)	1.025 (0.577, 1.826)	0.931
	6	22	0.386 (0.078)	9	0.155 (0.064)	0.402 (0.176, 0.845)	0.021
	12	20	0.351 (0.077)	2	0.034 (0.024)	0.098 (0.016, 0.336)	0.002
Anal discomfort	0	22	0.386 (0.105)	14	0.241 (0.079)	0.625 (0.313, 1.210)	0.170
	6	24	0.421 (0.109)	2	0.034 (0.024)	0.082 (0.013, 0.276)	0.001
	12	24	0.421 (0.109)	0	0 (0)	——	——
Diarrhea	0	48	0.842 (0.203)	37	0.638 (0.125)	0.758 (0.491, 1.160)	0.204
	6	63	1.105 (0.237)	15	0.259 (0.100)	0.234 (0.128, 0.399)	<0.001
	12	53	0.930 (0.222)	7	0.121 (0.061)	0.130 (0.054, 0.267)	<0.001
Dehydration	0	42	0.737 (0.165)	19	0.328 (0.111)	0.445 (0.253, 0.753)	0.003
	6	42	0.737 (0.165)	3	0.052 (0.038)	0.070 (0.017, 0.193)	<0.001
	12	41	0.719 (0.166)	0	0 (0)	——	——

IR: incidence rate; SE: standard error; IRR: incidence rate ratio; CI: confidence interval. ^a^ *p* value obtained via Poisson regression.

**Table 4:** Generalized Mixed-Effects Model Analysis of the Impact of *B. infantis* on Gastrointestinal Symptoms (*p* values).

	Poor Appetite	Nausea	Vomiting	Stomachache	Anal Discomfort	Diarrhea	Dehydration
Model 1							
week	0.463	0.371	0.425	0.219	0.777	0.126	0.666
group	<0.001	0.005	0.002	0.001	<0.001	<0.001	0.001
week × group	1.000	0.999	0.246	0.094	0.999	0.233	0.910
Model 2							
week	0.464	0.371	0.425	0.253	0.777	0.126	0.666
group	<0.001	<0.001	0.004	0.007	0.001	0.001	<0.001
Model 3							
week	0.464	0.371	0.489	0.182	0.777	0.130	0.666
group	<0.001	<0.001	0.016	0.010	0.004	0.001	<0.001

Model 1: adjusted for baseline values, time, group, and interaction terms comprising time and group. Model 2: additionally adjusted for delivery and primary caregiver to correct for differences between groups. Model 3: additionally adjusted for breastmilk and vegetable intake during the intervention.

**Table 5:** Effect of YLGB-1496 on Biochemical Parameters in Stool Samples from Study Participants (Mean ± SD).

Variables	Control	YLGB-1496	*p * ^a^	Model ^b^	*p * ^c^		
					Week	Group	Week × Group
pH							
0	6.26 ± 1.31	6.70 ± 1.15	0.066	1	0.476	0.072	0.246
6	6.54 ± 1.19	7.06 ± 0.91	0.009	2	0.476	0.117	
12	6.58 ± 1.09	6.87 ± 0.78	0.100	3	0.476	0.263	
FC (mg/g)							
0	1.30 ± 0.71	1.33 ± 0.59	0.776	1	0.123	0.137	0.595
6	1.02 ± 0.74	1.48 ± 0.85	0.003	2	0.122	0.114	
12	1.42 ± 2.75	1.67 ± 1.44	0.537	3	0.122	0.225	
AAT (mg/g)							
0	0.28 ± 1.03	0.41 ± 0.47	0.399	1	0.251	<0.001	0.580
6	0.09 ± 0.11	0.42 ± 0.76	0.001	2	0.251	<0.001	
12	0.11 ± 0.12	0.48 ± 0.62	<0.001	3	0.251	0.008	

SD: standard deviation, FC: fecal calprotectin, AAT: α1-antitrypsin. ^a^ *p* value obtained via Student’s *t*-test. ^b^ Model 1: adjusted for baseline values, time, group, and interaction terms comprising time and group; Model 2: additionally adjusted for delivery and primary caregiver to correct for differences between groups; Model 3: additionally adjusted for breastmilk and vegetable intake during the intervention. ^c^ *p* values for the intervention were obtained from the linear mixed model.

## Data Availability

The data are not publicly available due to ethical restrictions.The data presented in this study are available on request from the corresponding author.
